# The Role of SDF-1/CXCR4/CXCR7 in Neuronal Regeneration after Cerebral Ischemia

**DOI:** 10.3389/fnins.2017.00590

**Published:** 2017-10-24

**Authors:** Xi Cheng, Huibin Wang, Xiuchun Zhang, Shanshan Zhao, Zhike Zhou, Xiaopeng Mu, Chuansheng Zhao, Weiyu Teng

**Affiliations:** ^1^Neurology, The First Hospital of China Medical University, Shenyang, China; ^2^Geriatrics, The First Hospital of China Medical University, Shenyang, China

**Keywords:** stromal cell-derived factor-1, CXCR4, CXCR7, neurogenesis, ischemic stroke

## Abstract

Stromal cell-derived factor-1 is a chemoattractant produced by bone marrow stromal cell lines. It is recognized as a critical factor in the immune and central nervous systems (CNSs) as well as exerting a role in cancer. SDF-1 activates two G protein-coupled receptors, CXCR4 and CXCR7; these are expressed in both developing and mature CNSs and participate in multiple physiological and pathological events, e.g., inflammatory response, neurogenesis, angiogenesis, hematopoiesis, cancer metastasis, and HIV infection. After an ischemic stroke, SDF-1 levels robustly increase in the penumbra regions and participate in adult neural functional repair. Here we will review recent findings about SDF-1 and its receptor, analyse their functions in neurogeneration after brain ischemic injury: i.e., how the system promotes the proliferation, differentiation and migration of neural precursor cells and mediates axonal elongation and branching.

Chemokines are a family of small proteins with chemotactic properties capable of attracting immune cells; they are classified into four subfamilies CXC, CC, C, and CX3C according to the relative situation of the first two of four cysteins. Stromal cell-derived factor-1 (SDF-1) as a CXC subfamily chemokine is bone marrow stromal origin, also known as CXCL12. The SDF-1 mRNA can translate into six protein isoforms: SDF-1α, SDF-1β, SDF-1γ, SDF-1δ, SDF-1ε, SDF-1ϕ, are translated from, all possess the same first three exons but different fourth exon (Murphy et al., 2000), most of related studies focused on SDF-1α at present due to its abundant and extensive expression in all organs (Gleichmann et al., 2000; Yu et al., 2006). CXCR4 was previously considered as the only SDF-1 receptor until the identification of CXCR7 (RDC1) in T lymphocytes (Balabanian et al., 2005; Burns et al., 2006). SDF-1 and its receptors are involved in various physiological and pathological processes, as well as the development of nervous system, including inflammatory processes, neurogenesis and angiogenesis in the central nervous system (CNS). Here, we provide a summary of the current knowledge focusing on the role of SDF-1/CXCR4/ CXCR7 in neuroplasticity in the normal and injured brain.

## Expression of SDF-1/CXCR4/CXCR7 in the intact CNS

Expression of SDF-1 can first be detected in 12.5 d embryos (E12.5 d). During the embryonic stage, SDF-1 is expressed in the developing cortex, ventricular peripheral zone with very high expression levels in the meninges. After birth, SDF-1 is constitutively expressed in the brain stem, olfactory bulb, hippocampus, hypothalamus, cerebellum, and blood vessels of the brain. The high-level expression of SDF-1 in the hippocampus lasts throughout the organism's lifetime. In the mature CNS, SDF-1 is known to be constitutively expressed in neurons, glial cells, endothelial cells, and meningeal cells (Berger et al., 2007; Tiveron and Cremer, 2008; Cui et al., 2013).

Similar to the expression of SDF-1, the expression of CXCR4 can be detected during early development i.e., on E8.5 d, during full embryonic stages, it is expressed mainly in the ventricular zone (VZ) and marginal zone (MZ; Tissir et al., 2004). The postnatal expression of CXCR4 becomes gradually reduced in the above areas, but CXCR4 persists for the entire lifelong in dentate gyrus subgranular zone (SGZ) and olfactory bulb. Furthermore, CXCR4 is constitutively expressed in mature neurons, astrocytes, microglia, and ependymal cells (Banisadr et al., 2002; Stumm et al., 2002).

CXCR7 mRNA expression can be first observed on E11.5 d. During the embryonic stage, CXCR7 is mainly distributed in the developing cortex, VZ, SVZ, granule cell layer of cerebellar, dentate gyrus, caudate nucleus, and ganglion eminence. After birth, the expression of CXCR7 declines rapidly and at P7 only a few signals can be detected (Schönemeier et al., 2008a). In the adult brain, the expression of CXCR7 is normally maintained at a relatively low level, being detectable in the cortex, hippocampus, olfactory bulb, MZ, SVZ/IZ, cerebellum, hypothalamus, thalamus. If one utilizes cell type markers, then it seems that CXCR7 mRNA is expressed in neurons, endothelial cells, meningeal cells, astrocytes, and oligodendrocyte progenitor cells (OPCs; Thelen and Thelen, 2008).

## SDF-1/CXCR4/CXCR7 signaling pathways

SDF-1 binds to CXCR4 activates multiple G protein-coupled pathways, e.g., both Gαi and Gβγ subunits can trigger phosphatidylinositol-3-kinase (PI3K) and its main downstream targets Akt, mitogen activated protein kinase (MAPK) as well as the nuclear transcription factor NF-κB pathway; these properties have been described in astrocytes, neuronal progenitors (Bajetto et al., 2001; Wu et al., 2009; Cui et al., 2013). The Gβγ subunit triggers the activation of phosphatidylinositol-4,5-bisphosphate (PIP2), diacylglycerol (DAG), and inositol triphosphate (IP3) by phospholipase C (PLC) and mobilization of Ca^2+^. The increased levels of calcium activated proline-rich tyrosine kinase (PYK2), which can induce the activation of ERK1/2(Bajetto et al., 2001). In addition, CXCR4 also inhibits some cAMP pathways through the Gi component of GPCRs as demonstrated in primary cultures of neurons (Liu et al., 2003). Moreover, after SDF-1 binding, CXCR4 induces the activation of the Janus kinases JAK2 and JAK3 and the initiation of the STAT signaling pathway (including STAT1, 2, 3, and 5 subtypes) via the Gαi protein (Vila-Coro et al., 1999). All of these signaling pathways are known to be associated with cell proliferation, differentiation, migration, survival, and apoptosis. Finally, SDF-1/CXCR4 has been shown to induce GRK phosphorylation and β-arrestin activation, which in turn promotes the internalization and desensitization of CXCR4, thereby preventing the receptor from continually interacting with the G protein (Kumar et al., 2007). The interaction between SDF-1 and CXCR4 can be inhibited by AMD3100; this compound has been widely used as a pharmacological tool to reveal the involvement of CXCR4 and SDF-1 in various settings (De Clercq, 2010). However, it has been claimed that AMD3100 can function as an CXCR7 allosteric agonist, recruits β-arrestin to CXCR7, this property should be taken into consideration in future experiments (Kalatskaya et al., 2009). Human β-defensin3,CX549, T140 are novel antagonists of CXCR4, acting to competitively inhibit SDF-1 binding to CXCR4 (Feng et al., 2006; Xue et al., 2014; Wu et al., 2017).

**Figure 1:**
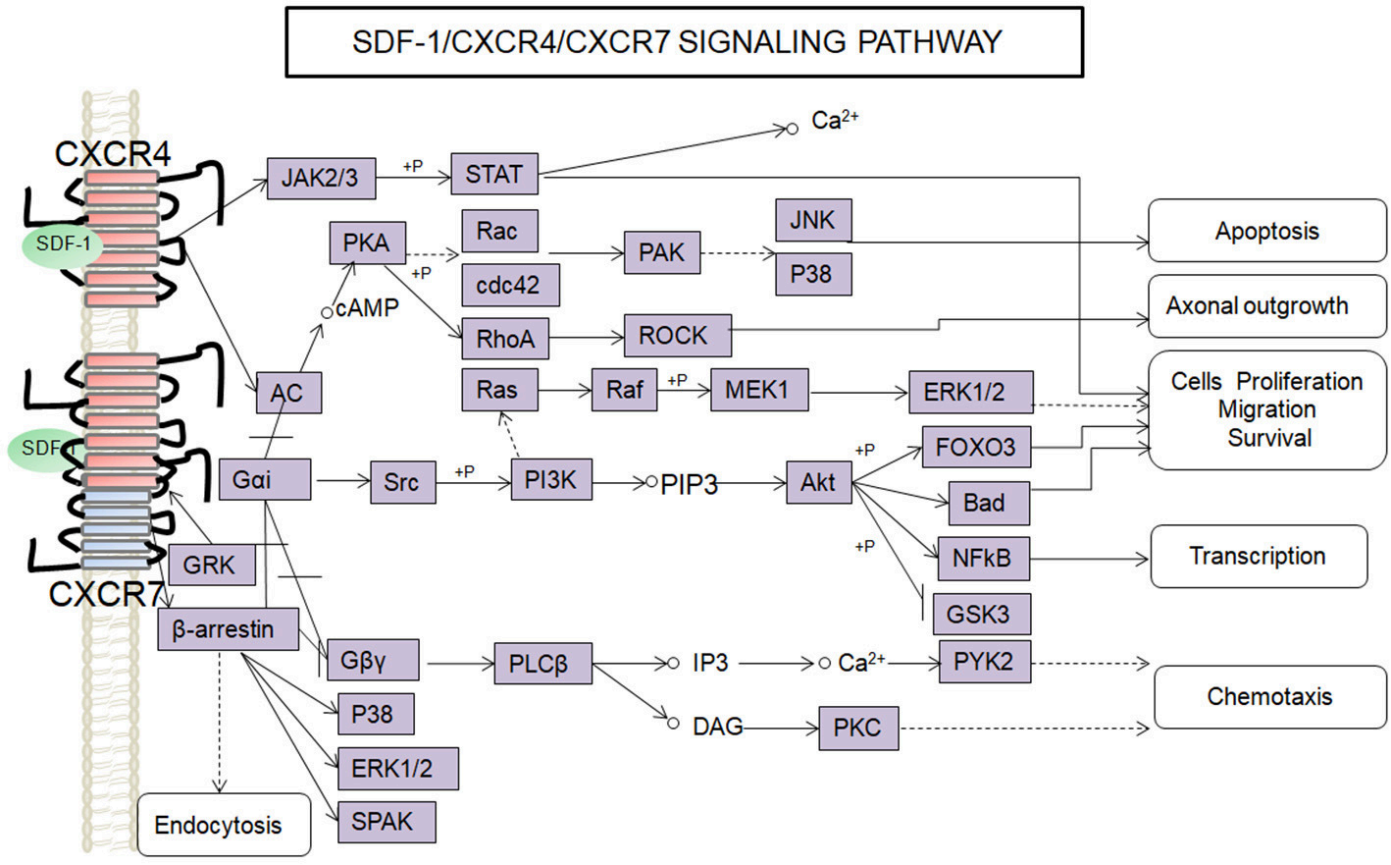
Overview of signaling pathways of the SDF-1/CXCR4/CXCR7 network. 
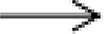
,
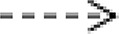
, 
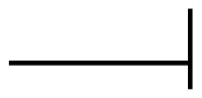
,+P indicate processes involving in direct promoting effect, indirect promoting effect, inhibitory effect, and phosphorylation effect, respectively.

Although, CXCR7 is a G protein-coupled receptor (GPCR), it does not trigger classical G-protein mediated signaling and the typical chemokine-induced Ca^2+^ release (Levoye et al., 2009). CXCR7 functions as a scavenger receptor for its cognate ligand and in that way can regulate the extracellular availability of SDF-1(Naumann et al., 2010). CXCR7 can form heterodimers with CXCR4 to form a structural trigger of the downstream signaling pathway (Puchert and Engele, 2014). CXCR7 and CXCR4 heterodimers induce the constitutive recruitment of β-arrestin and the subsequent activation of extracellular signal-regulated kinases-MAPK, including the ERK1/2, p38, and SAPK pathways (Décaillot et al., 2011; Sanchez-Martin et al., 2013). There are reports suggesting that activation of these pathways might be involved in cell survival and migration (Rajagopal et al., 2010; Wang et al., 2011b; Zhu et al., 2012; Guyon, 2014). In addition, SDF-1 has been claimed to induce the activation of ERK1/2 and AKT mediated by CXCR7 in astrocytes and Schwann cells. It was reported that SDF-1 induced the activation of protein kinase C (PKC) ξ/λ in astrocytes, and p38 in Schwann cells through CXCR7 (Odemis et al., 2010). Interesting, an *in vitro* study revealed that CXCR7 might be able to activate Gi/o proteins in primary astrocytes in the absence of CXCR4 and to induce Ca^2+^ release (Odemis et al., 2012) (Figure 1). Further studies will be required to determine whether CXCR7 binding to SDF-1 activates G protein-coupled pathways and to clarify the discrepancies observed between different cell types.

## Interaction between SDF-1/CXCR4/CXCR7

### CXCR7 controls the concentration of SDF-1 by mediating SDF-1 internalization

It has been estimated that affinity of SDF-1 for CXCR7 is almost 10 times higher than for CXCR4 (Balabanian et al., 2005; Zhu and Murakami, 2012). In this respect, CXCR7 can reduce the extracellular concentration of SDF-1, i.e., it primarily mediates SDF-1 internalization, after which it is subjected to lysosomal degradation (Boldajipour et al., 2008; Sanchez-Alcaniz et al., 2011; Wang et al., 2011b). This phenomenon is thought to clear excess extracellular SDF-1 molecules in order to keep the SDF-1 concentration at an optimal level to form the chemokine gradient required for cell migration. Interestingly, it was reported that high ligand concentrations reduced total cell surface expression of CXCR7, while SDF-1 does not affect the receptor internalization regardless of ligand level, indicating that high concentrations of SDF-1 inhibit the CXCR7 cycling system (Luker et al., 2010).

### CXCR7 regulate the expression of CXCR4 and its downstream pathways

CXCR7 may promote internalization of CXCR4 by forming heterodimers with CXCR4, most of the CXCR4 is degraded inside the cells, whereas CXCR7 is recycled back to the cell membrane (Naumann et al., 2010; Zhu et al., 2012). Consistently, CXCR7 agonists were able to reduce the level of CXCR4, resulting in a reduction of the cells' sensitivity to SDF-1 (Uto-Konomi et al., 2013). However, CXCR7 knockdown lead to increase significantly in the levels of extracellular SDF-1, which in turn triggered nearly 70% CXCR4 endocytosis and degradation, which causes detect of CXCR4-mediate functions (Sanchez-Alcaniz et al., 2011). This is convincing evidence that the presence of CXCR7 maintains a stable expression level of CXCR4 and ensures its sensitivity to SDF-1. CXCR7 can form heterodimers with CXCR4 to regulate SDF-1/CXCR4 downstream signaling processes. For example, CXCR7 and CXCR4 heterodimers can enhance β-arrestin-dependent signaling pathways (i.e.ERK1 / 2, P38MAPK, SAPK) and inhibit Gi signaling pathway, (Sierro et al., 2007), reducing the SDF-1 dependent inhibition of cAMP production in HEK293 cells (Décaillot et al., 2011). However, CXCR7 were also reported to weaken the Gαi activation and calcium signaling mediated by CXCR4 (Levoye et al., 2009). Thus, it seems that CXCR7 can regulate both the levels of CXCR4 and the downstream pathways normally activated by SDF-1/CXCR4.

### Co-expression of CXCR4 reduces the expression of CXCR7 and its affinity of SDF-1

Similar effects were also observed when one examines the CXCR4 regulation of the SDF-1/CXCR7 axis. The affinity of SDF-1 for CXCR7 reduces when there is the expression of CXCR4 at the cell surface (Burns et al., 2006). The coexpression of CXCR4 and CXCR7 enhanced β-arrestin recruitment for CXCR7, even in the absence of SDF-1 (Décaillot et al., 2011). The detailed mechanisms underlying the interactions of SDF-1/CXCR4/CXCR7 are complicated, but do seem to involve the following two processes: (i) CXCR7 removes excess extracellular molecules of SDF-1 and facilitates CXCR4 internalization; this reduces cellular responsiveness to SDF-1 (Petit et al., 2007). Co-expression of CXCR7 and CXCR4 attenuates the ability of CXCR4 to interact with G proteins. (ii) Co-expression of CXCR4 enhances the recruitment of CXCR7 by β-arrestin, and reduces the affinity of SDF-1 for CXCR7. Thus, the CXCR4 and CXCR7 seem to be kept in balance by mutually regulating their expressions and signaling pathways.

## Role of SDF-1/CXCR4/CXCR7 in neurogenesis

In mammalian CNS, these are two regions continually producing new neurons, namely SVZ and the SGZ of the dentate gyrus. The SVZ is a thin layer of cells on the side wall of the lateral ventricles, consisting of three major cell types: neuroblasts (type-A cells) astrocytes neural stem cells (type-B cell) and transient amplifying cells (type-C cells). These cells migrate down the rostral migratory stream (RMS) under the direction of proinflammatory cytokines into the olfactory bulb (OB), where they switch to radial migration toward their final destination. The specific role of SVZ derived newborn neurons has been associated with odor discrimination and olfactory information processing (Gheusi et al., 2000; Imayoshi et al., 2008; So et al., 2008; Breton-Provencher et al., 2009). The neuroprogenitor cells (NPCs) in the SGZ of the DG are divided into type 1 and type 2 according to their morphology and specific molecular markers. These cells proliferate and differentiate into dentate granule cells (DGCs), and these newly generated DGCs continually integrate into the mature hippocampal network (Fukuda et al., 2003).

**Figure 2:**
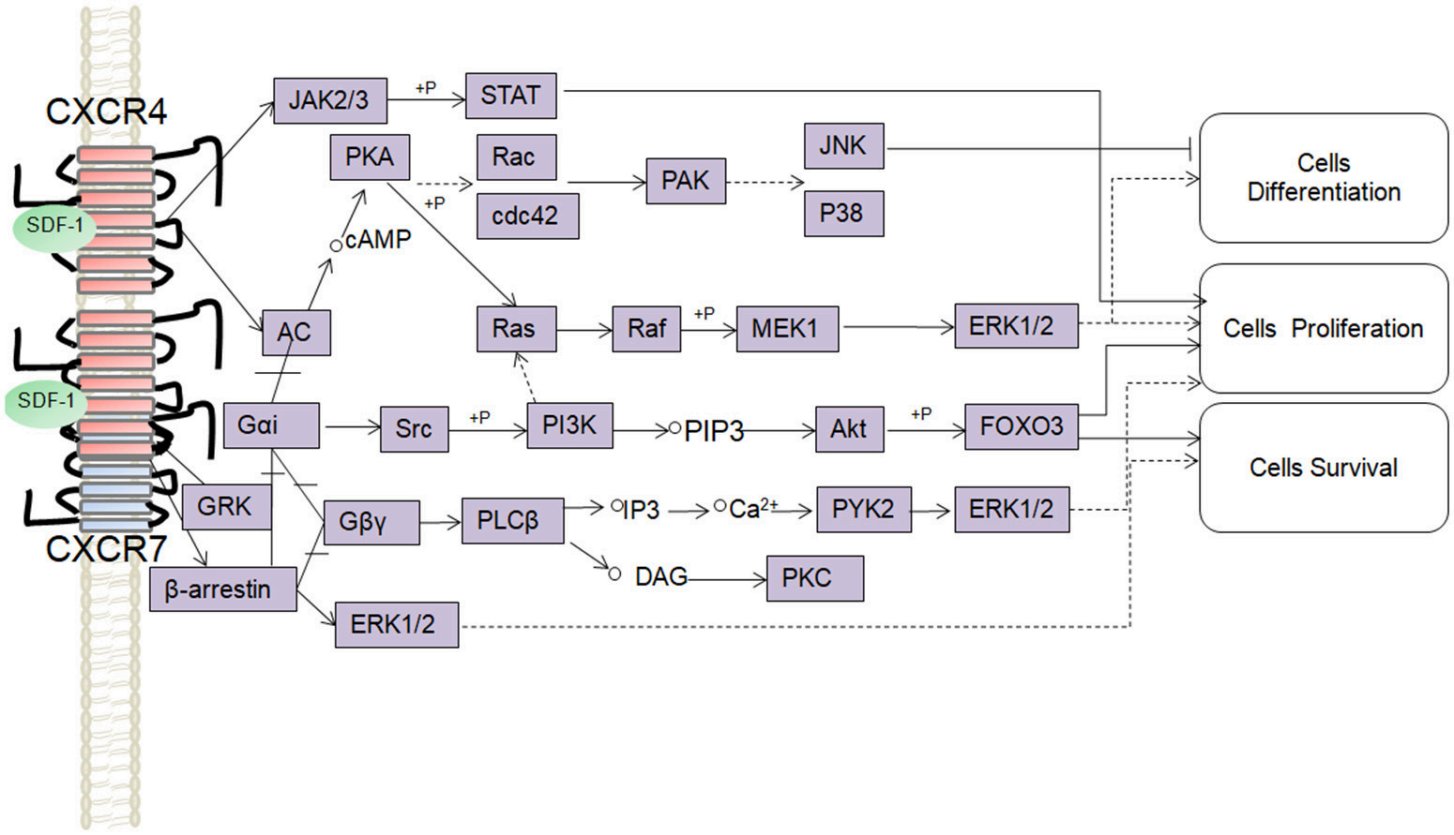
Schematic overview of SDF-1/CXCR4/CXCR7 interactions and their effects in neurogenesis.

The SDF-1/CXCR4/CXCR7 system plays an important role in the process of neurogenesis by influencing the migration, proliferation and differentiation of NPCs (Figure 2).

### The SDF-1/CXCR4 signaling mechanisms induce NPCs migration

In the early stages of development, SDF-1/CXCR4 participates in the migration and morphogenesis of DG granule cells. It has been shown that DG morphogenesis is significantly disrupted in the absence of CXCR4, an effect primarily attributable to disturbances in the migration of the DG precursors to their targets (Lu et al., 2002; Li et al., 2009). *In vivo* studies in postnatal mice have reported the distribution of cells expressing CXCR4 in brain to be consistent with the location of NPCs, evidence that CXCR4 was involved in SDF-1-induced migration of NPCs (Tran et al., 2007). It was claimed that SDF-1/CXCR4 increased the radial migration of NPCs from the SVZ/RMS toward the infarct areas in a dose-dependent manner (Dziembowska et al., 2005), and this was mediated via the PI3K/Akt, MAPKs, and JAK2/STAT pathways (Holgado et al., 2013; Chen et al., 2014; Merino et al., 2015). In all above signaling pathways, SDF-1α modulate migration speed of NSCs through ERK1/2 and p38 signaling pathways, while Akt and JNK signaling pathways are involved in the migration directional of NSCs (Chen et al., 2014). *In vitro* studies utilizing the allergy toxin C3a indicated that SDF-1α-induced the migration of the brain-derived NPCs by regulating the phosphorylation of ERK1/2. Interestingly, C3a promoted SDF-1α-induced migration of NPCs at low concentrations, whereas it inhibited the migration of NPCs in the presence of high concentrations of SDF-1α (Shinjyo et al., 2009).

### Several observations emphasize the complex role of SDF-1/CXCR4 in NPCs proliferation

Numerous studies have demonstrated that SDF-1 can promote the proliferation of NPCs with SDF-1 concentrations ranging from 10 to 350 ng/ml via PI3K,ERK1/2, Akt-1, and its downstream target FOXO3a pathway in conjunction with the JAK/STAT pathways (Gong et al., 2006; Wu et al., 2009; Zhu et al., 2017). However, there are studies demonstrate diverse results, Krathwohl et al. reported that when CXCR4 was activated by SDF-1 this maintained NPCs in a quiescent state and promoted the survival but not the proliferation of cultured NPCs (Krathwohl and Kaiser, 2004). A recent study demonstrated that SDF-1 alone did not promote the self-renewing and proliferation of NPCs, but regulated the expansion and proliferation of NPCs induced by growth factors, such as fibroblast growth factor/epidermal growth factor (bFGF/EGF; Li et al., 2011). However, Liu et al. reported overexpression of CXCR4 in the absence of SDF1α decreased neural progenitor cell proliferation (Liu et al., 2008). These discrepancy may attribute to the heterogeneous nature of NPCs, which depends on the age of the embryo and adulthood, and reflect the multiple functions of SDF-1/CXCR4 in the process of NPCs proliferation, but the details of CXCR4 signaling remains uncertain.

### SDF-1/CXCR4 promotes NPCs differentiation in a dose-dependent manner

The SDF-1/CXCR4 system also plays a crucial role in the differentiation of NPCs. When cultured NPCs from adult mice were exposed to low (20 ng/ml) or high (100–500 ng/ml) concentrations of SDF-1 there was a 2.3-fold increase in the expression of neuron-specific enolase-2 (NSE2, a neuronal marker) and microtubule-associated protein 2 (MAP 2ab, an axonal and dendritic marker) positive neurons. In addition, SDF-1α induced F-actin polymerization and reorganization, form some rigid cytoskeletal structures. These results support the idea that SDF-1 directly stimulates NPCs to differentiate into neurons *in vitro* (Shinjyo et al., 2009; Chen et al., 2014). *In vitro* experiments showed that MAPK signaling pathways were associated with NSCs differentiation, ERK1/2 phosphorylation is an early signaling event required for the neuronal differentiation of NSCs (Li et al., 2006), whereas another study demonstrated that inhibition of JNK facilitated NSC differentiation, which was negatively correlated with the differentiation of NSCs (Yang et al., 2005). Interestingly, during the development of NPCs, endogenous SDF-1, and CXCR4 have different expression locations. The expression of CXCR4 is significantly increased when NPCs differentiate into neurons, whereas the expression of SDF-1 is upregulated when NPCs differentiate into astrocytes. This is consistent with the proposal that CXCR4 is highly expressed by NPCs and neurons whereas astrocytes are the main source of SDF-1 in the brain (Peng et al., 2007).

### SDF-1/CXCR4 provides neurons with a survival advantage both *in vivo* and *in vitro*

CXCR4 signaling promotes the survival of NPCs during CNS development. In CXCR4-knockout mice, the number of NPCs in the neurosphere outgrowth was only half that present in wild-type mice (Dziembowska et al., 2005). Several *in vitro* studies have demonstrated that CXCR4 activation by SDF-1 induces neuronal survival through Akt phosphorylation and FOXO3a activation (Khan et al., 2003, 2008). Besides, treatment with a novel CXCR4 antagonist CX549 can increase the survival of neurons through inhibiting microglia activation and promote the behavioral recovery. These studies suggest that SDF-1/CXCR4 has a dual effect on the neuronal survival.

### SDF-1/CXCR7 also regulates the NPCs migration independent of CXCR4 or interactions with CXCR4

There is evidence indicating that CXCR7 participates in the migration of NPCs (Sanchez-Alcaniz et al., 2011; Chen et al., 2015; Merino et al., 2015). The migration of NPCs could be inhibited by either antagonists of CXCR4 or CXCR7, suggesting that the migration of the NPCs is dependent on the integrated function of both CXCR4 and CXCR7 (Merino et al., 2015). Furthermore, NPCs in which there is CXCR4 knockout can still migrate to SDF-1, and this phenomenon can be blocked by CCX771 (an antagonist of the CXCR7 receptor), evidence that CXCR7 can mediate the migration of NPCs independently of CXCR4(Liu et al., 2013; Chen et al., 2015). Several studies have reported that CXCR7 mediated the migration of primordial cells by shaping the extracellular SDF-1 gradient during neuronal development (Dambly-Chaudière et al., 2007; Boldajipour et al., 2008; Sanchez-Alcaniz et al., 2011). Cells must become polarized before their migration, i.e., there has to be the formation of a lamellipodial protusion at the leading edge of the cell involving a reorganization of the cytoskeleton (Ridley et al., 2003). Chen et al. revealed that CXCR7 colocalized with Rac1 at the leading edge of the cell, especially in the lamellipodial protrusion (Chen et al., 2015). A similar increased interaction was observed between β-arrestin and CXCR7 after SDF-1 treatment. These results support the proposal that the activation of both Rac1 and β-arrestin are required to promote the CXCR7-mediated migration of NPCs (Chen et al., 2015).

### SDF-1/CXCR7 regulates the NPCs proliferation independent of CXCR4

Wang et al. reported that SDF-1 pretreatment can induce the transition of G0/G1 phase to the S phase, which is crucial for proliferation of cells. CXCL12 treatment also increased the expression of β-catenin and CyclinD1, CyclinD1 as a gene plays a vital role in the cell proliferation process. The proliferation of mNPCs mediated by SDF-1 can be blocked in CXCR7-defect mNPCs, whereas CXCR4 defect mNPC did not significantly attenuate SDF-1-mediated mNPCs proliferation, these results indicate that CXCR7 can regulate proliferation of NPCs independent to CXCR4 (Wang et al., 2016).

### The survival-supporting and anti-apoptosis effects of SDF-1/CXCR7 for NPCs

The property of CXCR7 to support the survival of NPCs mediated has also been reported. For example, there was a significant elevation in the amount of SDF-1 secreted by the human CD133-derived multipotent mesenchymal cells after a stroke, and this promoted the survival of NPCs. Bakondi et al. also demonstrated that only CXCR7 was associated with the survival of NPCs induced by SDF-1; they came to this conclusion by inhibiting either CXCR4 or CXCR7 pharmacologically. This conclusion was at odds with previous findings. Therefore it will be important to determine whether complete blockade of CXCR4 receptor can achieve similar results after a stroke. Zhu et al. reported CXCLl2-induced endocytosis signaling mediated by CXCR4 and that CXCR7 sustained activation of downstream ERKl/2 signaling in endosomes and these effects could protect NPCs from undergoing apoptosis (Zhu et al., 2012). In conclusion, CXCR7 and CXCR4 may play overlapping but different roles in modulating the survival of NPCs.

## SDF-1/CXCR4/CXCR7 and anatomical neural rewiring in the adult CNS

It has been demonstrated that newly generated neuroblasts expressing NeuN migrate from the SVZ to their destination, where they form synaptic connections integrate into the existing neuronal network. It is necessary for the formation of neural circuits that these cells detect the correct location of the neurons in order to build connection between axons, as these are processes which are crucial not only in the development of the CNS but also in functional repair after brain injury. The formation of neuronal circuits involves several cellular events: migration of the cells, axonal arborization, axonal guidance, axonal elongation, and branching (Pujol et al., 2005).

Evidence is accumulating that SDF-1 is involved in axonal pathfinding, outgrowth, and branching (Arakawa et al., 2003; Chalasani et al., 2003). In the early development of hippocampal neurons, CXCR4 accumulates in the leading processes of the NPCs. The expression of CXCR4 is less extensive but it is widely distributed along the axons and dendrites in mature neurons (Pujol et al., 2005). Studies have shown that SDF-1 signaling influences the dynamic morphology of migrating interneurons, which include branching frequently and locate their soma, these branches may serve as sensors capable of detecting surrounding guidance cues. It has been claimed that SDF-1 can reduce the branching frequency and increase the speed of migrating interneurons, to avoid these interneurons being attracted away from the SDF-1-enriched migratory paths (Pujol et al., 2005). However, it is thought that SDF-1 signaling within interneurons reduce as they exit the migration streams (López-Bendito et al., 2008). This reduction in SDF-1 signaling increases the branching of the leading interneurons which, in turn, slows the speed of migration, allowing migrating interneurons to sense cortical guidance cues and arrive at their ultimate destination. The process is dependent on the CXCR4-coupled G protein which inhibits the cAMP pathway, resulting in activation of the Ras-Akt pathway (Arakawa et al., 2003; Pujol et al., 2005). In this model, SDF-1 affects pathfinding by modulating cell morphology, not necessarily involving its actions as an attractant or a mitogen (Li et al., 2008; Lysko et al., 2011).

Numerous studies have shown that there is a process of axonal regeneration in the mammalian CNS after injury (Benowitz and Carmichael, 2010; O'Donovan, 2016). Axonal sprouting occurs from the intact motor cortex into the peri-injured cortex and striatum, as well as the corticospinal tract (CST) originating from the intact side of the motor cortex into the denervated region of the cervical spinal cord. A correlation between the extents of functional recovery and somatotopic reorganization of the non-lesioned hemisphere has been described in several studies (Dancause et al., 2005; Lindau et al., 2014; Wahl et al., 2014). However, most of the sprouting axons in this process do not participate in the functional recovery since there is an intrinsic failure to reach a regenerative state partly because of the non-supportive local microenvironment. Several intrinsic myelin-associated proteins including Nogo-A, myelin-associated glycoprotein (MAG), oligodendrocyte-myelin glycoprotein (Omgp), and the glial scar formed at the lesion site limit the possibilities for axonal growth and plasticity (Silver and Miller, 2004; Yiu and He, 2006; Berry et al., 2008; Benowitz and Carmichael, 2010; Pernet and Schwab, 2012). *In vitro* cell culture experiments have revealed that the presence of CNS myelin fragments can inhibit axonal outgrowth by combining with the Nogo receptor (NgR) and the activated Rho/Rho-kinase signaling pathway, RhoA and its target, the Rho-associated coiled-coil protein kinase (ROCK), which promotes the actin cytoskeleton reorganization and growth cone collapse (Domeniconi and Filbin, 2005; Benowitz and Carmichael, 2010; Pernet and Schwab, 2012; Fujita and Yamashita, 2014).

**Figure 3:**
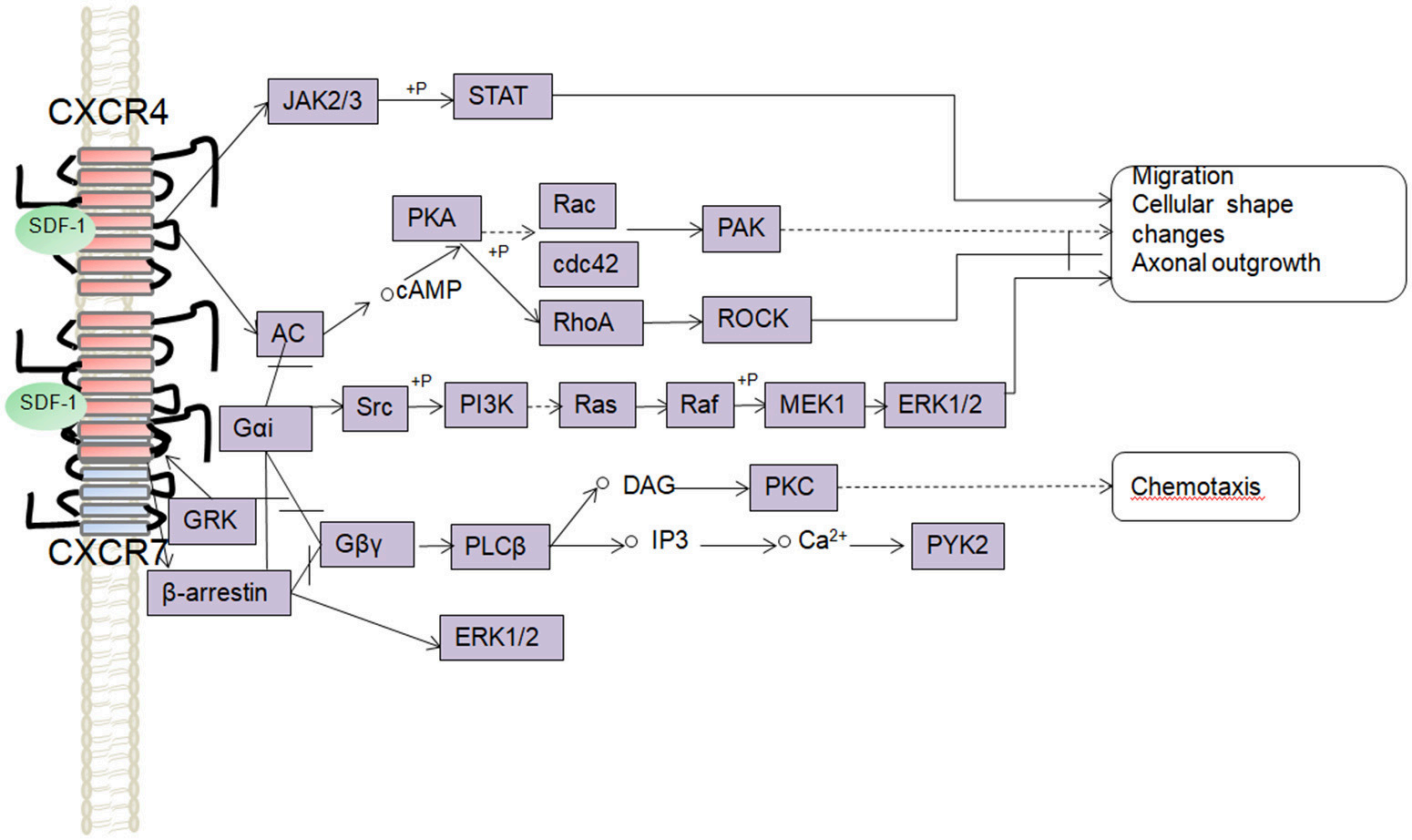
Schematic overview of signaling pathways involved in the SDF-1/CXCR4/CXCR7 mediated axonal outgrowth, cells migration, and chemotaxis.

It has been speculated that the significant expression of CXCR4 in the growth cone is related to its catalytic role in axonal elongation and branching (Pujol et al., 2005). A local intrathecal infusion of SDF-1 resulted in neurite outgrowth and axonal spouting after a spinal cord injury (SCI). The sprouting of corticospinal tracts (CST) probably involves a signaling pathway involving CXCR4 and CXCR7, both have be detected in the CST axons (Opatz et al., 2009; Jaerve et al., 2011). *In vitro* experiments have confirmed that SDF-1/CXCR4 can increase the levels of intracellular cAMP through multiple G protein-coupled pathways, thus inducing Rho inactivation, and promoting axonal elongation (Wang et al., 2008). In line with these reports, Arakawa et al. observed that SDF-1 could induce axonal elongation via the Rho/mDia pathway in cerebellar granule neurons (Arakawa et al., 2003). Furthermore, guanosine-5′-triphosphate (GTP) Rho kinase-Rac has been also reported to promote axonal growth and cytoskeletal formation (Bakondi, 2011). It has been speculated that SDF-1/CXCR4 might activate the Rac and Rho/mDia pathways to promote axonal regeneration at low SDF-1 concentrations, whereas at high concentrations, it would inhibit axonal outgrowth through the Rho/ROCK pathway (Arakawa et al., 2003; Pujol et al., 2005). In addition, the intracellular cAMP levels elevated after SDF-1/CXCR4 signaling subsequently could induce an up-regulation of arginase I, the enzyme which catalyzes the production of polyamines. Thus, over-expression of arginase I or administration of polyamines can inhibit the effects of MAG and myelin (Cai et al., 2002). Unlike CXCR4, the CXCR7 receptors are more evenly distributed in neurons, but the downstream pathways behind their role in neurite outgrowth are not completely understood. In a mouse model of multiple sclerosis (MS), the administration of a CXCR7 antagonist prevented inflammatory damage to axons, preserving the axonal integrity and improving the behavioral recovery (Cruz-Orengo et al., 2011). Liu et al. found that SDF-1 may change the cellular cytoskeleton by increasing the polymerization of actin filaments, thus resulting in preserved neurite outgrowth, a process mediated via phosphorylation of ERK1/2 after its activation by SDF-1/CXCR7 signaling (Liu et al., 2013) (Figure 3). However, clarification of the role of CXCR7 in axonal outgrowth will require more work in the future, perhaps conducted in animal models with conditional knockout or RNA silencing of receptors.

## Remyelination impact of SDF-1/CXCR4/CXCR7

Oligodendrogenesis i.e., the formation of new oligodendrocytes (OLGs) happens during development, physiological conditions in the adult brain but also after a stroke (de Castro et al., 2013). Since injured OLGs are unable to produce a new myelin sheath (Franklin and Ffrench-Constant, 2008; McTigue and Tripathi, 2008; Zhang et al., 2013), and mature OLGs do not proliferate in the adult brain (Itoh et al., 2015), newly generated OLGs are essential for axonal repair and remyelination after a stroke. Generally, OPCs serve as the main source of remyelinating cells (Tripathi and McTigue, 2007). Therefore, enhancement of endogenous oligodendrogenesis in ischemic brain will be a new therapy target to facilitate brain repair processes and reduce neurological deficits. Overall, the maturation of OPCs into mature OLGs experience several stages, including pro-OLGs, immature OLGs, and mature OLGs. Different stages can be defined by the multiple specific cell-surface receptors: i.e., A285,PDGFα for oligodendrocyte-type 2 astrocyte (O-2A) cells, O4 for pro-OLGs, O1 for immature OLGs and MBP, PLP, MAG, CNPase for mature OLGs (Butts et al., 2008; Arai and Lo, 2009).

During development, OPCs originate in the VZs of the brain and spinal cord and migrate along the neural tube throughout the entire CNS before their differentiation into myelinating OLGs (de Castro et al., 2013; Bergles and Richardson, 2015). OPCs has been verified the most dispersive migratory cells in the entire oligodendroglial lineage. Studies showed that migration of OPCs required the vasculature as a physical brace (Tsai et al., 2016). At E12, OPCs stream was observed to migrate away from the medial ganglionic eminence in association with vascular scaffold. Many of these migratory OPCs are located along blood vessels with their cell bodies directly on the abluminal endothelial surface. *In vivo* study showed that Wnt activation drived OPCs to the vascular scaffold and inhibited dispersion of OPCs normally into CNS parenchyma. While one of the most highly up-regulated factors in Wnt activated OPCs was CXCR4, which is a direct Wnt target expressed by the endothelium in developing brain. Furthermore, treatment with AMD3100 led to a reversal of OPC clustering to vascular scaffold, which demonstrated a Wnt activated, CXCR4-dependent mechanism of OPCs associated with vascular scaffold. Beyond that, CXCR4 was expressed by OPCs during embryonic period but was down-regulated along with Wnt down-regulation in differentiating mature OLGs, thus, Wnt activation and CXCR4 signaling was involved in blockage of the OPCs differentiation (Tsai et al., 2016).

Under normal conditions, SVZ-Type B cells slowly divide and can give rise to Olig2-expressing type C cells, which develop into highly migratory OPCs. In turn, these cells continue to divide locally or they differentiate into myelinating cells (Maki et al., 2013). These new generated OPCs can migrate to the corpus callosum (CC), fimbria fornix, striatum, septum, and gray matter in brain and spinal cord where some of OPCs differentiate into mature oligodendrocytes (Menn et al., 2006).

There is a great deal of research demonstrate that both NSCs and OPCs participate in the remyelination of the white matter injury. SVZ-derived NSCs would shift into oligodendroglial lineage cells into the damage area in corpus callosum. Moreover, OPCs adjacent to the damaged area may also be more important when the demyelination occurs in these areas (de Castro et al., 2013). After brain ischemia, recent studies demonstrate that OPCs originating from neural progenitor cells in the SVZ of the lateral ventricle differentiate into myelin forming oligodendrocytes under physiological and ischemic conditions. The OPCs need to pass through the following phases in order to achieve remyelination (McTigue and Tripathi, 2008): OPCs go through a switch from a quiescent state to a regenerative state. During this activation step, microglia and astrocytes become activated and generate proliferative factors that induce reactively activation of OPCs to the myelin injury (Glezer et al., 2006). Subsequently, OPCs migrate while still undergoing proliferation into the regions surrounding the lateral ventricles and the infarcted areas, this is called the recruitment phase of remyelination (Larsen et al., 2003). Following recruitment, the OPCs differentiate into mature oligodendrocytes and remyelinated axons. The secretion of certain chemokines, such as SDF-1, produced at the lesion site is a critical component of the CNS injury response, ensuring that OPCs migrate and mature appropriately to replace damaged cells (Franklin and Ffrench-Constant, 2008). Although, there is a large number of OPCs in the brain, the remyelination is very limited, which is attributable to several reasons, i.e., the presence of oligodendrocyte differentiation inhibitors in the environment, which originate from astrocytes, demyelinated axons or myelin debris (Kotter et al., 2006; Wang et al., 2011a), the inhibition of gliascar, and the lack of OPCs or preOLGs differentiation into myelin forming OLGs. However, in the primary demyalinating disease MS, remyelination is abundant enough to partially recover lesions and ameliorate the symptoms at some degree. Demyelination would be present in the ischemic brain before the acute ischemia happens, particularly in the normal aging (Pannese, 2011). For this reason, remyelination may play a more important role in cerebral ischemia than we have recognized.

Expression of SDF-1 is increased in neuronal nuclei (NeuN+) neurons, glial fibrillary acidic protein (GFAP+) astrocytes and ionized calcium binding adaptor molecule (Iba+) microglia cells in the mouse brain after MCAO; this response peaked at 1 d, and began to decline at 3 d, and at 14 d it was detected only in neurons and microvessels. At 5 w after the ischemia, the expression of CXCR4 was abundant in cells displaying both platelet derived growth factor receptor alpha (PDGFRα+, a marker for OPCs) and neuron-glial antigen (NG2+, a marker for OPCs) OPCs (Li et al., 2015). In line with the above findings, CXCR4 protein levels were significantly upregulated in NG2+OPCs at 6 weeks after a SCI (Bracchi-Ricard et al., 2013). Li et al. suggested that CXCR7 would be only expressed in mature myelin after MCAO (Li et al., 2015). Study has supported the beneficial effect of SDF-1/CXCR4 on OPCs proliferation and migration. Li et al. reported adeno-associated virus (AAV)-SDF-1 gene injection into the ischemic perifocal area could enhance MBP expression and the numbers of PDGFα+ cells and PDGFα+/BrdU cells in SVZ and perilesional areas after MCAO, while coadministration of AMD3100 could block the beneficial effect of SDF-1 in protecting myelin sheath integrity and promoting OPCs proliferation. Furthermore, CXCR7 could be observed on MBP+ mature myelin sheaths rather than OPCs, which might suggest that CXCR7 plays a role in OPCs maturation (Li et al., 2015). At present, the evidences of the role of SDF-1/CXCR4/CXCR7 in remyelination mainly focus on demyelinating disease, especially in MS models, there are few studies on cerebral ischemia and brain injury. All these researches may provide some valuable hints on the function of SDF-1/CXCR4/CXCR7 in remyelination. Knowledge of the precise expression of SDF-1/CXCR4/CXCR7 in OLG cells and specific mechanism in remyelination is required to further explore.

Growth factors including FGF-2, IGF-1, VEGF, and BDNF contribute to CNS development, they also are expressed in cortex after brain ischemia and involved in neuronal regeneration, remyelination and angiogenesis process. One of the factors is especially under focus in the last years: FGF-2, since it was described as present only in the histopathological scenarios where remyelination succeeds (Clemente et al., 2011). A number of data have been published to reveal the real role of FGF-2 in normal myelination and remyelination after CNS injury (de Castro et al., 2013). FGF-2 could induce remyelination in MS models by promoting OPCs migration (Bribián et al., 2006; de Castro et al., 2013), increasing the numbers of type B cells in the adult SVZ, OPCs and oligodendrocytes in the demyelinated areas (Azim et al., 2012), decreasing the numbers of CD8+ T cells and macrophages/microglia, and ameliorating nerve fiber degeneration and axonal loss (Rottlaender et al., 2011). These studies focus on the chronic stage of EAE. FGF2 also promote remyelination in cerebral ischemia models, bFGF exerted a protective effect on myelin by increasing the myelin thickness, the number of myelinated axons, and myelin basic protein expression in the hypoxia-ischemia (HI)-induced demyelinated neonatal rat corpus callosum. *In vitro* research also confirmed bFGF ameliorated the impaired mitochondria and cell processes induced by oxygen/glucose deprivation (OGD) to promote the survival of isolated O4-positive preOLs (Qu et al., 2015). The relevance of FGF-2 has been put in value together with other relevant factors, such as ligands of CXCRs in the CNS injury based on its active function in remyelination with complex mechanism.

## SDF-1/CXCR4/CXCR7 and cerebral ischemia

After focal cerebral ischemia, SDF-1 is up-regulated and released within 24 h after focal cerebral ischemia by astrocytes, microglia, and vascular endothelial cells in damaged areas (Ceradini et al., 2004; Hill et al., 2004). The level of SDF-1 peaks at 7 d and remains elevated until 16 w. It has been reported that activation of hypoxia inducible factor 1 (HIF-1) can increase the expression of SDF-1 in endothelial cells, with the effect being proportional to the extent of the reduction in oxygen tension (Ceradini et al., 2004). In rodents, at 2 weeks after the injury, newly-generated NPCs migrate from the SVZ through a pathway similar to RMS to the ischemic boundary region (Kokaia and Lindvall, 2003; Lindvall et al., 2004; Williams et al., 2014). This migration can last for several months after a stroke (Thored et al., 2006). These SVZ-derived neuroblasts can differentiate into neurons, oligodendrocytes and astrocytes and replace damaged neurons. It is noteworthy that CXCR4 is expressed in the NPCs and the actions of the SDF-1/CXCR4 complex are critical for the migration of NPCs to SDF-1-riched injury sites after cerebral ischemia (Imitola et al., 2004; Thored et al., 2006; Shyu et al., 2008). Blockade of the CXCR4, did not reduce the number of NPCs but disrupted the migration of NPCs, leading to a failure of the newborn neurons to localize to the ischemic tissue (Ohab et al., 2006; Thored et al., 2006). *In vitro* studies have revealed that the expression of CXCR7 is increased at 24 h after hypoxia (Liu et al., 2013). In the cerebral ischemia boundary, the expression of CXCR7 increases rapidly; while it is scarce in the ischemic area (Schönemeier et al., 2008a,b). The distributions of CXCR7 suggest that it may mediate SDF-1 internalization; in this way it could create a low to high SDF-1 concentration gradient from the cerebral ischemia boundary to the insult core to guide the migration NPCs to the damaged area. In summary, CXCR4 and CXCR7 may play different roles in NPCs recruitment to ischemic brain tissue.

It is evident that neurogenesis is not the only phenomenon involved in the functional recovery after stroke. In addition, stroke-induced angiogenesis has been shown to enhance post-stroke neurogenesis (Jin et al., 2006; Thored et al., 2007) (Zhang et al., 2009). Angiogenesis consists of multiple steps, including the mobilization and proliferation of BMCs, the structural remodeling of vessels, proliferation of perivascular, and the formation of capillary vessels (Hossmann, 2006). SDF-1 plays an important role in recruitment, proliferation and maturation of endothelial progenitor cells (EPCs) which express CXCR4 in the ischemic boundary zone (IBZ). The increased expression of SDF-1 in plasma is clearly related to the increased number of EPCs present after a stroke (Bogoslovsky et al., 2011). There is conclusive evidence that SDF-1 is involved in the recruitment of EPCs to the injury region by binding to their cell surface CXCR4 receptors (Petit et al., 2007; Zheng et al., 2007; Shiba et al., 2009; Shen et al., 2011). *In vitro* studies have revealed that SDF-1 can promote the proliferation of EPCs and inhibit the apoptosis of EPCs (Shao et al., 2008). SDF-1/CXCR4 may be involved in the regulation of the proliferation and migration of smooth muscle cell (SMC) progenitors which are required for growth and maturation of capillaries and arterioles (Schober and Zernecke, 2007). Collectively, these results suggest that SDF-1 displays promising potential to promote angiogenesis and improve outcome after ischemic stroke, and therefore it can be viewed as a new therapeutic target for treating cerebral ischemic.

## Conclusions

SDF-1/CXCR4/CXCR7 have recently attracted significant attention due to their important role in adult neurogenesis not only by promoting the migration, proliferation and differentiation of NPCs but also mediating axonal elongation and branching as well as supporting remyelination. There is now convincing data indicating that SDF-1/CXCR4/CXCR7 can promote neurogenesis and angiogenesis, two interdependent processes required for recovery after a stroke. Therefore, a better understanding of how SDF-1 is expressed and functions would be beneficial in efforts to develop specific therapeutic strategies after ischemic stroke. Although, much is known about SDF-1, there are still a great many unanswered questions. For example, clarification of the different downstream pathways being activated in different cell types when SDF-1/CXCR4/CXCR7 in different processes. More studies *in vitro* will be required to unravel the complex role of SDF-1. One fascinating approach would be to try to amplify the role of the SDF-1/CXCR4/CXCR7 system in the mature CNS. One can speculate that SDF-1/CXCR4/CXCR7 signaling pathways may become new therapeutic targets since they hold the possibility of promoting a functional recovery after a stroke.

## Author contributions

XC has drafted the manuscript; HW and XZ have provided editing and writing assistance; SZ, ZZ, and XM have revised it critically for important intellectual content; CZ and WT have approved the final version to be published. All persons who have made substantial contributions to this manuscript.

### Conflict of interest statement

The authors declare that the research was conducted in the absence of any commercial or financial relationships that could be construed as a potential conflict of interest.
